# Seventh Annual Session of the Maryland State Dental Association

**Published:** 1890-04

**Authors:** 


### SEVENTH ANNUAL SESSION OF THE MARYLAND STATE DENTAL ASSOCIATION.

(Continued from page 181.)

The annual session was held on December 5 and 6, 1889, at the St. James Hotel, Baltimore, the president, E. P. Keech, M.D., D.D.S., in the chair.

*Thursday, December 5, 1889.—Evening Session.*

DISCUSSION OF DR. WM. A. MILLS'S PAPER ON "TOMATO-POISONING."

*Dr. Grindall.*—Mr. President, I think our friend, Dr. Mills, is mistaken in ascribing the cause of the trouble to which he refers as being due to the tomato. It will probably be found that it was due to calomel, though the difficulty may have been aggravated by the acid of the tomato. I have known of cases in which patients have been afflicted in the way spoken of by Dr. Mills, but the cause in those cases was properly attributable to calomel.

*Dr. E. Nelson.*—I desire to ask Dr. Mills whether he has determined, by chemical analysis, the peculiar acid in the tomato which affected the teeth in the manner he has described, and why it is that only the teeth of which he has spoken and not the teeth of other persons were affected?

*Dr. Mills.*—That is a conundrum to which I can only answer, "I give it up." In the statement I have made I have endeavored simply to state actual facts. I anticipate, at some future day, to give a history of the discovery of this disease. I may add here that the conditions of which I have spoken have, for years, been very marked in cases among young people. I have treated patients for the trouble, but did not know at the time what the exact cause was, and I only discovered it by accident. I have myself lost teeth from this very disease; the specimen I have exhibited here being a tooth from my own mouth. I assume that for that reason I am better able to judge of the cause of the trouble than are others who have not had personal experience with it. I lost two teeth in experiments upon myself, and that is why I claim to speak with some authority. Possibly every member present has had more or less experience in contending with cases of this kind, without supposing that the cause was to be found in the tomato. I have watched very closely the developments in my own case. While on the floor, I may say that this is only one of many cases of a like character to which my attention has been called. I propose to detail, at some future day, the results of my observations in other directions,—viz., quinine and other poisons.

As I intimated at the outset, I do not propose at this time to pronounce any decision upon the phenomena, but simply to throw out the suggestions that have occurred to me, with a view to the matter being taken up later by some one more capable than myself of reaching a determination upon it.

*Dr. T. S. Waters.*—I do not rise to discuss this subject, but merely to refer Dr. Mills to our friend, Dr. Rawlings, of Lexington, Ky., who has written considerable on the disease, claiming that the original cause of it is pyorrhœa alveolaris. By corresponding with Dr. Rawlings, I suggest that Dr. Mills may acquire more scientific data than he has now to enable him to reach the bottom of the trouble in what he has spoken of as the tomato disease.

*Dr. D. Genese.*—The statement of Dr. Mills and the condition of the tooth exhibited by him, which shows a speck upon it, leaves the matter plain to my mind as to the cause of the disease being other than the one he has stated. I happened to come from a country in which the tomato is a rarity, being almost as rare as the peach, and yet I found there evidences of the same action on the teeth which I have seen here. Since residing in this country I have known people who were accustomed to eat the tomato in its raw state, as taken from the vine during the season after ripening, instances of that kind coming under my notice throughout a period of twelve years, and yet I have not observed any erosion of the teeth from that cause. In a district which I have been visiting occasionally for the last year or so, and which is a great tomato-growing district, there has been serious trouble with the teeth, but the cause of it has not been attributed to tomato-eating. I visited some of the cottages there on matters of business other than dentistry, and, while there, I found that the attending physician had prescribed for the trouble with the teeth from three to ten grains of calomel, to be taken several times a day. You may imagine the effect of this upon the teeth.

I think that Dr. Mills will find, on close investigation, that the tomato, as an article of food, is one of the most healthful vegetables we have, and that, if eaten when ripe and in proper condition, the acid from it is no more destructive to the teeth than is the juice of an apple, the pear, or any other fruit in ordinary use. It is well known that the strawberry has a very acrid, pungent juice; and its effect on the teeth is somewhat peculiar. It will whiten the teeth, but if you insert in the mouth a paper test, five or ten minutes after a quantity of strawberries have been eaten, you will not find any acid remaining. The same thing may be said of other fruits from which considerable acid is derived and from which no injurious effect upon the mouth is apparent.

Before reaching any conclusion on the subject, I suggest that we await the results of further investigations, made on a more scientific basis than were those which have been stated; and the results we may possibly receive at our next meeting.

*Dr. Mills.*—I simply wish to put on record the actual facts in regard to the remarkable conditions to which I have called attention, so that, in the course of time, when the matter is again brought up, it may be known that these facts have been ascertained and stated. In doing this, I would say that I am not surprised that the theory I have advanced has met with a good deal of criticism. Indeed, I almost expected to hear it said, after what I have alleged as having happened in my own experience, that I had filled myself with calomel from the crown of my head to the soles of my feet. I know that such an inference may be drawn from what has been said. But I also know that there are medical authorities who state that the action of the tomato on the teeth is exactly the same as that of calomel. I also know, from what I have read on the subject, that certain facts have been demonstrated in regard to the effects produced by tomato eating. It is stated that, since the introduction of canned tomatoes into the empire of China (about two-thirds of all the tomatoes put up in the United States are consumed there), cancer, a disease that was comparatively little known there, has increased to an extent of from forty to fifty per cent. This is all attributed to the eating of canned tomatoes. By some it is attributed to the action of the muriatic acid on the solder, forming a lead salt. Nevertheless, it is our duty to meet facts as we find them. We have a practical one here. We should keep our eyes open and deal with these facts fairly and without any preconceived notions of our own. If the cause of the trouble is in the tomato, let us say so; if it is not, let us ascertain that fact.

*Dr. R. Grady.*—I understand Dr. Mills to state that he wants to go on record as having discovered the disease known as tomato-poisoning.

*Dr. Mills.*—Yes, sir.

*Dr. Grady.*—But Dr. Waters has stated that this matter is already on record; that it has already been written upon by Dr. Rawlings.

*Dr. Mills.*—With the permission of Dr. Grady, I would like to ask, through the chair, if Dr. Waters can inform me as to the date at which the writings referred to make their appearance.

*Dr. Waters.*—They appeared in the *Southern Dental Journal* some five or six years ago, and were in reply to articles written by Dr. Riggs, of Hartford, Conn., in connection with pyorrhœa alveolaris. Dr. Rawlings took very strong grounds in support of the theory that tomatoes were the original cause of pyorrhœa.

*Dr. Genese.*—The theory of tomatoes causing pyorrhœa must be regarded as exploded, because pyorrhœa existed in Europe, particularly in England, long before the tomato was introduced there.

*Dr. Mills.*—Allow me one moment. My statement was that I simply wished to go on record as to what I say. Of course, if the gentleman named has written on this subject, he antedates my statement by two years.

*Dr. Grady.*—By five years.

*Dr. Mills.*—If it is claimed that he antedates me by five years, I would call attention to the fact that I have been talking on this question and speaking to my friends upon it for three years. Now, I would like to speak upon the treatment of pyorrhœa alveolaris in general.

Some eight or nine years ago I had a case of a female patient who was troubled with what is now known as pyorrhœa alveolaris. The treatment of the patient embraced the usual remedies,—viz., Robinson's remedy, iodine and carbolic acid, the chisel, the gouge, and chemical compounds. The success of this treatment seemed to be uncertain, as the patient returned year after year, and I was almost ready to abandon my efforts in despair of effecting a cure in the case, which was only one of many like cases. For a period of twelve months all traces of her were lost. At the expiration of that interval she returned to have a tooth filled. Remembering the young lady's case, I examined her mouth to ascertain what ravages the disease had made, when, to my surprise, I found that all symptoms of pyorrhœa alveolaris had disappeared. I questioned her closely as to the remedy she had been using. She informed me that, having noticed that a cousin of hers in Washington, whom she was visiting, had beautiful teeth and healthy gums, she learned that her relative, and the sisters of her relative also, had healthy mouths; they for years had been using as a tooth-powder carbonate of magnesium. Upon their advice she had, for twelve months, been using the same. She was conscious of the fact that her gums had become healthy and her teeth firm in their sockets, but her improved condition did not specially attract her attention as it did mine. I reflected upon the case and concluded that, if the fact was as she stated it, the carbonate of magnesium must have made the cure. I then began to experiment with the drug and continued my experiments for several years. First I used it as a dentifrice, and directed patients to rub their gums with it at night, before retiring. Observation showed that from six to twelve months were required to effect anything like a change for the better except in some few cases, which responded promptly in six or eight months. Even in cases in which a patient would not submit to the operation of having the deposits removed from around the necks of the teeth it acted promptly. After further reflection upon a method of improving the preparation for use as a dentifrice, I tried boric acid in combination with carbonate of magnesium in the proportion of from one-half to one drachm of the acid to one ounce of the carbonate of magnesium, with sufficient winter-green to flavor. This was intended to act as a powerful germicide and to prevent fermentation. And the preparation has invariably given the utmost satisfaction, my patients returning after an interval of forty-eight hours and assuring me that they had experienced the greatest relief.

While I do not assert positively that the application of these remedies has, in every instance, effected a perfect cure, I am able to say, upon the assurances of friends to whom I have given the formula, and who have personally witnessed the results of its application, that the action of it has been almost miraculous in producing a cure. Dr. McDonald told me that with him the remedy worked most satisfactorily. He did not strictly follow my directions, but used the formula for injections into the pockets while patients were at his office, and he expressed himself as being astounded by the improvement which resulted. So far as my own experience has gone, I can say that I think we have in these chemicals that which, if it does not effect a thorough cure, will certainly ameliorate the disease.

*Dr. Genese.*—Mr. President, I had the pleasure of listening to to Dr. Mills on this same subject some three or four years ago; and I am able to say that I have given the preparation referred to by him a thorough trial in every sense of the term, and have found it wanting. It has no effect upon teeth affected by pyorrhœa other than that which plain water would have. The carbonate of magnesium is insoluble. It will glide over the deposits upon the teeth and leave them just as they were before. In pyorrhœa we have an insidious disease that lies far below the surface. The preparation produced by Dr. Mills will not cure that disease, for the reason that the disease lies at a point beyond that which the preparation reaches.

I am indebted to Dr. Adair of the Southern Dental Association for an idea contained in an article in one of the numbers of the *Southern Dental Journal*, and which I have put in practical operation and have found to be exceedingly valuable in practice. I cannot remember the date when it appeared, but the substance of the article is that he advises, in these cases of pyorrhœa, and particularly where we have that condition in which pus is oozing around the gum margin, to flatten a piece of platinum, bringing it to a nice shape, then taking away all but the point, and leaving the shaft so fine that it will not injure any part with which it comes in contact. This is then heated somewhat, but not too hot. It is then dipped down around the teeth, between the cementum and the gum-lining. You thus cause the destruction of the tissue, though only to a very slight extent. Dr. Adair claims that the reaction in the deposit, the fresh tissue in the place of that which is destroyed by the cautery, will cure the pyorrhœa. Not very long ago I saw a practical demonstration of this. I have tried it in two cases, and they have succumbed entirely to the treatment. One of them was that of a long-standing fistula of the superior maxilla, with pyorrhœa extending from the central incisor to the first bicuspid. All the other teeth were gone. The entire treatment was confined within a period of about three weeks, when the patient reported himself as cured.

*Dr. Mills.*—I am rather surprised to hear Dr. Genese make the statement which he has just made. He is on record as having, on several occasions, recommended carbonate of magnesium for pyorrhœa alveolaris. In another place he is on record as recommending boric acid, carbonate of magnesium, and chalk. As this preparation was originally suggested by myself, and as I have heretofore spoken about it, I thought that Dr. Genese would give me the credit for it, just as he has given credit to Dr. Adair for his preparation for the treatment of pyorrhœa. I am surprised that he should come here to-night and say that he had found it to be unsuccessful and of non-effect, particularly as I stated at the outset of my remarks that, with the magnesia alone, an interval of from six to twelve months was required before any decided effect or improvement would be shown.

I believe that magnesia is a splendid thing to use in these cases, and my belief has been strengthened since I have been reading on the subject. I believe, too, that pyorrhœa alveolaris, so called, is nothing more nor less than an anæmic condition of the tissues surrounding the teeth, and that magnesia (having an affinity for iron in the blood) carries the blood corpuscles to the parts and the surrounding tissues and builds them up, producing a healthy action and flow of blood. Boric acid, being a germicide and an antifermentative as well as the winter-green, simply adds more strength to the compound.

*Dr. Genese.*—I wish to say that I may have mentioned these preparations at the meeting of the Southern Association, or at one of the Northern meetings, simply as remedies untried. If my memory serves me, I made that statement shortly after the meeting at which Dr. Mills had recommended these preparations. As I have now tried them for three years, I am able to speak experimentally. I believe you all know the action of magnesia, and I will ask you, as scientific men, whether, in your opinion, magnesia is going to have any effect upon the lining membrane of the gum tissues or on the teeth when they are attacked by pyorrhœa, as claimed by Dr. Mills. It is known to you that we must first have mechanical action to remove the deposit, and then we must have some pungent preparation or antiseptic to do away with the pus formation.

*Dr. A. J. Volck.*—I desire to mention a case of a lady, a native of Bermuda, who was in this city on a visit during the summer and remained during the following winter. She came to my office some time in September. Hers was one of the most pronounced cases of what is called pyorrhœa alveolaris. It occurred to me at the time to try the remedy which Dr. Mills had spoken of once in our meeting. I sent to that gentleman for the prescription, and he very kindly gave me a copy. I applied that remedy in the case of this lady, and continued to apply it for about seven weeks, when the treatment proved to be entirely successful. I was profoundly impressed with what I saw of the promptness with which the disease yielded to this treatment. That case is the only one in which I have seen this treatment made use of. I have no doubt there are cases in which it may be used successfully.

The lady remained here for some three or four weeks after the use of the remedy was discontinued, and I saw no unfavorable change in her condition and no indication of a relapse. She supplied herself with the preparation for her future use in the event of a return of the trouble. I directed the patient to rub it into the gums and around the teeth, particularly in the evening, before she retired, and to allow it to remain there. I also instructed her to use it as a tooth-powder during the day, as she had opportunity.

*The President.*—Is the formula for that powder the one that has been stated by Dr. Mills?

*Dr. Volck.*—The formula is the same.

The discussion of the subject here closed.

